# Adolescent Mental Toughness Questionnaire (aMTQ10): development, validation and norms

**DOI:** 10.3389/fpsyg.2026.1661207

**Published:** 2026-06-23

**Authors:** Andrew Denovan, Kenneth Graham Drinkwater, Amanda Vernalls, Sarah Jenkins, Neil Dagnall

**Affiliations:** 1 School of Psychology, Liverpool John Moores University, Liverpool, United Kingdom; 2 School of Psychology, Manchester Metropolitan University, Manchester, United Kingdom; 3 Youth Sport Trust, Loughborough, United Kingdom

**Keywords:** adolescent, incremental validity, mental toughness, Mental Toughness Questionnaire (MTQ), norms, psychometric evaluation

## Abstract

**Introduction:**

Research has established that mental toughness (MT), the capacity to withstand stress and sustain performance under pressure, is affiliated with real-world outcomes such as psychological well-being and academic success. To balance the conceptual importance of MT with the practical constraints of assessing the construct within large test batteries, theorists developed the Mental Toughness Questionnaire 10 (MTQ10), an abridged 10-item version of the 48-item Mental Toughness Questionnaire (MTQ48). Despite using high school students in the development of the MTQ10, investigators failed to assess adolescent comprehension of the instrument.

**Methods:**

To address this, the present study validated an adolescent version (aMTQ10) of the scale. The authors used an expert panel to evaluate item accessibility and implemented age-appropriate modifications. Validation of the aMTQ10 derived from a sample of 5,305 UK secondary school pupils from 80 schools.

**Results:**

Confirmatory factor analysis (CFA) supported a one-factor model. Invariance existed across age and school type, with partial invariance observed for gender. Analysis of latent means found that boys scored higher than girls and 13–14-year-olds scored higher than 14–15-year-olds. Additionally, private school pupils scored higher than state school pupils. The aMTQ10 demonstrated good internal reliability and convergent validity. Confirming the instrument’s incremental validity, aMTQ10 scores uniquely predicted subjective well-being beyond belongingness.

**Discussion:**

These results indicate that the aMTQ10 is a psychometrically satisfactory adaptation of the MTQ10 suitable for educational settings. The established normative data enables educators and practitioners to accurately interpret age-related MT scores and support adolescent psychological development.

## Introduction

Mental toughness (MT) is increasingly recognized as an important psychological resource. Research robustly links MT to enhanced psychological wellbeing (e.g., Mojtahedi et al., 2021, 2023; Papageorgiou et al., 2019, 2023). It is also associated with improved academic achievement (e.g., Denovan et al., 2021a; Lin et al., 2017; Stamp et al., 2015). Although definitions of MT vary (see Gucciardi, 2017), there is broad agreement that its benefits arise from its role as a protective buffer against stress, enabling individuals to function effectively under pressure and cope with adversity (Dagnall et al., 2021).

Conceptually, MT is a non-cognitive skill rather than a form of intellectual ability. It reflects the possession of intrinsic and developed values, attitudes, emotions, and cognitions that facilitate goal achievement (Drinkwater et al., 2019; Gucciardi et al., 2009). These characteristics shape how individuals manage themselves and interact with others (Denovan et al., 2023a,b). In turn, they promote adaptive cognitive appraisals that influence how individuals approach, respond to, and evaluate pressure, challenge, and difficulty.

These properties manifest as a positive mindset that enables individuals to thrive under pressure, including managing both difficulty and success (Denovan et al., 2023a). This proactive quality distinguishes MT from resilience, which refers to the capacity to recover from adversity (Denovan et al., 2022). In contrast, MT reflects an active, forward-facing disposition rooted in its broader construct composition. Specifically, the hardiness components of commitment (i.e., involvement and persistence), control (perceived influence over events and outcomes), and challenge (viewing difficulties as opportunities for growth), alongside confidence (i.e., belief in one’s abilities and capacity to succeed) (Denovan et al., 2023a,b; Perry et al., 2021).

Clough operationalized these components within the 4C/6C model of MT. The 6C extension reflects the subdivision of control (life and emotional) and confidence (abilities and interpersonal) into distinct subscales. This framework informed the development of the 48-item Mental Toughness Questionnaire (MTQ48; Clough et al., 2002), which remains the predominant measure of MT. However, the length of the MTQ48 can limit its practical use in applied settings where time and participant resources are constrained (e.g., educational, occupational, and elite sporting contexts). To address this, researchers developed the abridged.

MTQ18 and MTQ10, the latter comprising the highest loading items across the 6C dimensions (Dagnall et al., 2019; Papageorgiou et al., 2018).

Although both abbreviated versions demonstrate acceptable psychometric properties, evidence indicates that the MTQ10 is the more robust unidimensional measure. Specifically, relative to the MTQ18, it exhibits superior model fit, stronger factor loadings, and greater predictive validity (i.e., is a better prognostic indicator of life satisfaction) (Dagnall et al., 2019). Subsequent cross-cultural evaluation has supported the structural validity of the MTQ10, while identifying minor item-level method effects and partial invariance across countries (i.e., UK, Greece, and Italy) (Denovan et al., 2024). Collectively, these findings position the MTQ10 as a concise and psychometrically sound measure of global MT.

The evaluation of short MTQ measures is important given their frequent use in research, particularly within educational contexts where MT is assessed alongside a range of demographic and psychological variables (e.g., Brand et al., 2015, 2017; Denovan et al., 2021a). The development of nationally adapted versions (e.g., Indonesian: Guntoro et al., 2025; Russian: Denovan et al., 2021b) further reflects the demand for brief, contextually appropriate instruments.

Despite these advances, the fact that researchers developed the MTQ measures using adult participants potentially limits their suitability for younger populations (Clough et al., 2002; Perry et al., 2013). This is particularly true in relation to adolescence because it is a sensitive developmental period characterized by rapid biological maturation, ongoing identity formation, and increased exposure to academic and social evaluation (Spear, 2000; Steinberg, 2005). During adolescence, individuals are negotiating greater academic demands alongside evolving peer relationships, heightened emotional reactivity, and increased autonomy (Eccles and Roeser, 2011; Smetana, 2010). These pressures make this stage especially relevant for the study of MT, as it is during this period that adaptive coping strategies and psychological resources are actively being formed and consolidated.

Higher levels of MT in adolescents have been associated with more effective stress management, improved emotional regulation, greater resilience in the face of setbacks, and more positive educational engagement and attainment outcomes (Gucciardi et al., 2009; Nicholls et al., 2011). Adolescence is also associated with increased vulnerability to psychological distress, heightened sensitivity to peer evaluation, and major educational transitions, all of which may intensify the importance of adaptive psychological resources such as MT (Sirsch, 2003; Putwain, 2008; Allen et al., 2024). Consequently, the development of age-appropriate measures capable of accurately assessing MT during this developmental period is important for both research and applied educational practice.

Cognizant of this, McGeown et al. (2018) developed the Mental Toughness Scale for Adolescents (MTS-A) for use within educational contexts. The MTS-A is an 18-item scale comprising six three-item subscales: Commitment, Challenge, Control (life), Control (emotions), Confidence (abilities), and Confidence (interpersonal). Analyses across two studies established the MTS-A as a valid and internally reliable psychometric instrument. Moreover, MTS-A factors were associated with positive adolescent outcomes, including higher academic motivation and engagement, greater well-being, and lower test anxiety.

Despite the existence of the MTS-A, researchers working with adolescents continue to rely on MTQ measures (MTQ48, MTQ18, and MTQ10) due to their dominance within the literature and extensive supporting psychometric evidence. However, because subsequent validation work has also predominantly relied on mixed and older samples (e.g., Perry et al., 2013, 2021), questions remain about the developmental applicability of MTQ measures. Importantly, the present study does not seek to replace the MTS-A, but rather to provide an adolescent-adapted version of the widely used MTQ10 framework, thereby facilitating continuity and comparability with the broader MTQ literature. Additionally, the brevity of the aMTQ10 may provide practical advantages within school-based research and large assessment batteries where administration time is constrained.

Although the MTQ10 was partially informed by research involving high school students (Papageorgiou et al., 2018; Dagnall et al., 2019), direct evidence regarding adolescent comprehension of MTQ items remains limited. This represents a critical gap in the literature, as it is unclear whether existing MTQ measures are interpreted by adolescents in the manner intended by their original adult-based development. Consequently, further work is required to ensure that item content is developmentally appropriate and that these measures validly capture MT within adolescent educational settings.

## The present study

Building on the age-related MTQ concerns, the authors drew on guidance from an expert panel of academics and youth researchers to perform age-appropriate modifications to MTQ10 items. This produced the Adolescent Mental Toughness Questionnaire (aMTQ10). To evaluate the psychometric properties of the aMTQ10, this study assessed its factorial structure, internal reliability, incremental validity, and measurement invariance. In addition, population norms were established to support interpretation of scores in adolescent samples.

Given the limited nature of item modifications, it was expected that the aMTQ10 would retain the correlated unidimensional structure of the MTQ10 and demonstrate high internal reliability. To assess incremental validity, the study examined whether the aMTQ10 predicted life satisfaction beyond an established measure of belonging. These constructs were selected because life satisfaction is a key indicator of subjective well-being, while belonging is a well-established predictor of adolescent adjustment and psychological health (Allen et al., 2024). Life satisfaction was also used as a criterion variable in prior MTQ10 validation work (Dagnall et al., 2019). By controlling for belonging, the study tested whether MT contributes unique variance in life satisfaction beyond social connectedness. This provides evidence that the aMTQ10 captures a distinct component of adolescent well-being rather than overlapping with related social constructs.

Invariance testing was conducted across gender (boys and girls), year group (Year 9 and Year 10), and school type (private and state). Consistent with findings for the MTQ10, it was anticipated that the aMTQ10 would demonstrate measurement invariance across these groups. Establishing invariance is essential to ensure that the construct is interpreted consistently across subgroups, supporting valid comparisons.

Finally, normative data were generated for the aMTQ10 to aid interpretation of scores within adolescent populations. These norms provide a reference framework indicating whether individual scores are low, average, or high relative to a school-based adolescent sample, thereby enhancing the practical and applied utility of the measure for both researchers and practitioners.

## Method

### Participants

The sample included 5,305 UK secondary school pupils (2,578 girls, 2,727 boys) from 80 schools (61 private/independent, 19 state). Participants comprised 2,865 Year 9 pupils (aged 13–14 years; 1,369 girls, 1,496 boys) and 2,440 Year 10 pupils (aged 14–15 years; 1,209 girls, 1,231 boys). Exact age data were not collected; therefore, school year was used as the relevant developmental grouping variable. Prior to analysis, 47 cases were excluded because of incomplete school identification information.

### Measures

#### Adolescent Mental Toughness Questionnaire

To adapt the MTQ10 for adolescent samples and produce the Adolescent Mental Toughness Questionnaire (aMTQ10), an expert panel comprising academics and youth researchers with expertise in adolescent psychology, education, and psychometrics reviewed item wording to identify language that may reduce accessibility for younger respondents. The panel included two psychology academics with experience in developing the MTQ10 and related psychometric measures of mental toughness and associated constructs, alongside two youth research professionals with substantial experience of conducting research and data collection with adolescent populations. The goal was to make minor developmental wording modifications that improved readability while preserving the conceptual meaning of the original MTQ10 items (Greenfield et al., 2013). Most changes involved simplifying phraseology rather than altering substantive content. For example, “Even when under considerable pressure I usually remain calm” became “Even when under lots of pressure I usually remain calm”, and “presented with several things to do at once” became “given several things to do at once”. Following revision, the panel reassessed items to ensure conceptual equivalence and developmental appropriateness.

When panel members made further suggestions about statement expression or clarity, content underwent iterative revision until panel members agreed that modified items aligned with the original and were consistent with adolescent comprehension. This procedure established the face validity of the adolescent Mental Toughness Questionnaire 10 (aMTQ10) (Macaskill and Taylor, 2010). Although modifications were intentionally minimal, developmental adaptation of psychometric measures remains important because seemingly small linguistic differences may affect comprehension, interpretation, and response validity in adolescent samples (Greenfield et al., 2013). Regarding response format, the aMTQ10 adopted the typical MTQ five-point scale (1 = “Strongly disagree” to 5 = “Strongly agree”). Supplementary Table S1 presents the original MTQ10 items alongside the adolescent-adapted wording used in the aMTQ10.

#### Sense of Belonging Scale

This study used a modified version of the Sense of Belonging Scale (SBS) (see Anderson-Butcher et al., 2012). This instrument captured students’ general perceptions of their relationship to their school in terms of connectedness. The SBS comprised five items (e.g., “I am accepted at my school”) and respondents answered via a five-point response scale (1 = “Not at all true” to 5 = “Really true”). The SBS has demonstrated acceptable psychometric properties (e.g., Anderson-Butcher and Conroy, 2002).

#### Satisfaction with Life Scale

The Satisfaction with Life Scale (SWLS) captures global cognitive judgments of life contentment (Diener et al., 1985). The instrument consists of five statements (e.g., “In most ways, my life is close to my ideal”). Participants rate their agreement with a seven-point Likert-type scale (1 = “Strongly disagree” to 7 = “Strongly agree”). The SWLS possesses good psychometric properties (Diener et al., 1985).

### Procedure

Data were collected using an online survey distributed through participating schools. Schools were recruited through existing educational networks and direct institutional contact. Participation was voluntary and no incentives were offered. Information sheets and consent procedures were provided to schools, parents/guardians, and pupils in accordance with institutional ethical approval procedures. Following survey completion, participants received a study debrief. The Ethics Manchester Metropolitan University Committee within the Faculty of Business and Law granted approval (Ethos ID #51727).

### Analysis

To assess the validity of the aMTQ10, the authors employed confirmatory factor analysis (CFA). CFA (Mplus v8; Muthén and Muthén, 2018) tested construct validity using a one-factor correlated uniqueness (CU) model based on Dagnall et al. (2019). Previous research has identified this as the optimal factor structure relative to competing models (Denovan et al., 2024). The CU model specified correlated residuals for negatively worded items (items 2 and 7; items 3 and 6) to account for shared wording effects.

Model fit was evaluated using chi-square, the Comparative Fit Index (CFI), Standardized Root-Mean-Square Residual (SRMR), and Root-Mean-Square Error of Approximation (RMSEA). Recommended thresholds for acceptable model fit are CFI ≥ 0.90, SRMR ≤ 0.08, and RMSEA ≤0.08 (Browne and Cudeck, 1992). Factor loadings ≥0.30 were considered satisfactory indicators of the latent construct (Gliner et al., 2016).

Measurement invariance analyses examined whether the aMTQ10 operated equivalently across gender (boys and girls), year group (Year 9 aged 13–14 and Year 10 aged 14–15), and school type (private and state). Configural, metric, and scalar invariance models were tested sequentially. Establishing invariance is important because it indicates that the scale measures the same underlying construct across groups, thereby supporting meaningful comparisons of scores. Following Chen’s (2007) recommendations, changes greater than 0.01 for CFI and 0.015 for RMSEA were considered evidence of reduced invariance. After invariance testing, latent mean comparisons were conducted. Reliability was assessed using McDonald’s omega and Cronbach’s alpha coefficients.

Incremental validity was evaluated using two complementary approaches. First, hierarchical regression examined whether the aMTQ10 explained additional variance in life satisfaction (SWLS) beyond sense of belonging (SBS), consistent with prior MTQ research. Second, structural equation modeling (SEM) was used because it accounts for measurement error, providing a more precise estimate of relationships among variables (Wang and Eastwick, 2020).

Following the SEM procedure outlined by Wang and Eastwick (2020), the first stage examined whether the aMTQ10 and SBS represented distinct constructs. This involved comparing a one-factor model with a two-factor model in CFA. Better fit for the two-factor model would indicate that the constructs are related but distinct. The second stage assessed whether the aMTQ10 uniquely predicted life satisfaction after statistically controlling for belonging. Lastly, analyses included the calculation of population norms.

## Results

### Factor structure

Skewness and kurtosis values fell within the acceptable range of −2 to +2 for all items (Field and Miles, 2010). The CU model exhibited satisfactory fit across indices, χ^2^ (33, *N* = 5,305) = 792.23, CFI = 0.93, SRMR = 0.04, RMSEA = 0.06 (95% CI of 0.06 to 0.07). Satisfactory factor loadings (>0.32) existed for all items (Table 1), and 50 % of items possessed loadings above 0.60, meeting Hair et al.’s (2010) stricter criterion. The average loading was 0.58.

**Table 1:** Descriptive statistics and parameter estimates (standardized factor loadings) of the aMTQ10.

Item number	Skewness	Kurtosis	Factor loading
Item 1	−0.45	−0.65	0.64
Item 2	0.73	−0.25	0.36
Item 3	−0.09	−0.83	0.48
Item 4	−0.56	−0.08	0.68
Item 5	−0.65	0.10	0.67
Item 6	0.13	−0.97	0.45
Item 7	0.15	−1.08	0.58
Item 8	−0.52	−0.12	0.72
Item 9	−0.71	0.39	0.52
Item 10	−0.40	−0.42	0.67

All loadings significant at *p* < 0.001.

### Multi-group invariance

Tests of invariance comparing gender (boys vs. girls) indicated no meaningful CFI or RMSEA change when comparing configural and metric models. However, the scalar model exhibited an unsatisfactory CFI difference (0.028). Freeing intercepts concerning items 2, 7, and 10 resulted in a satisfactory CFI change of 0.01. Computation of latent means with boys as the reference group indicated that females possessed significantly lower aMTQ10 scores, Δ*m = −*0.38, *p* < 0.001, *d* = 0.67.

Invariance analyses assessing school year (Year 9 vs. 10), and school type (private vs. state) revealed no major differences in CFI or RMSEA when progressing from tests of form to structure, and subsequently to intercepts (Table 2). Latent mean comparisons for school year (Year 9 as reference group) revealed that Year 10 scored significantly lower on the aMTQ10, Δ*m = −*0.03, *p* = 0.015, *d* = 0.06. Latent mean comparisons for school type (private as reference group) indicated that state school pupils possessed significantly lower aMTQ10, Δ*m = −*0.18, *p* < 0.001, *d* = 0.30. This indicates that comparisons between boys’ and girls’ latent mean scores should be interpreted cautiously at the item level, although overall comparisons of the underlying construct remain appropriate. The gender difference represented a medium-to-large effect (*d* = 0.67), whereas differences for school year (*d* = 0.06) and school type (*d* = 0.30) were small and small-to-medium, respectively.

**Table 2:** aMTQ10 invariance models.

Model	χ^2^	df	CFI	CFI difference	SRMR	RMSEA (90% CI)	RMSEA difference
Gender (girls vs. boys)							
Configural	804.66**	66	0.92		0.04	0.06 (0.06–0.06)	
Metric	820.15**	75	0.92	None	0.02	0.06 (0.05–0.06)	0.004
Scalar	1095.12**	84	0.90	0.028	0.05	0.06 (0.06–0.07)	0.006
Scalar (partial)	923.01	81	0.91	0.01	0.05	0.06 (0.05–0.06)	0.002
School year (Year 9 vs. Year 10)							
Configural	832.72**	66	0.93		0.04	0.06 (0.06–0.07)	
Metric	840.25**	75	0.93	None	0.04	0.06 (0.05–0.06)	0.004
Scalar	871.96**	84	0.93	0.002	0.04	0.05 (0.05–0.06)	0.003
School type (private vs. state)							
Configural	823.82**	66	0.93		0.04	0.06 (0.06–0.07)	
Metric	843.62**	75	0.93	0.001	0.04	0.06 (0.05–0.06)	0.004
Scalar	877.13**	84	0.92	0.002	0.04	0.06 (0.05–0.06)	0.002

χ^2^ = chi-square; df = degrees of freedom; CFI = Comparative Fit Index; SRMR = Standardized Root-Mean-Square Residual; RMSEA = Root-Mean-Square Error of Approximation. **p < 0.001.

### Reliability and convergent validity

Good reliability existed in relation to alpha and omega estimates (both 0.81). The aMTQ10 associated positively with SBS, *r* = 0.47, *p* < 0.001, representing a moderate positive association.

### Incremental validity

The hierarchical regression approach revealed in step 1 that SBS significantly positively predicted SWLS, *ß* = 0.71, *t* = 72.99, *p* < 0.001, with an R^2^ of 0.50. Entering aMTQ10 in step 2 significantly predicted SWLS, *ß* = 0.52, *t* = 47.52, *p* < 0.001, and accounted for additional variance (R^2^ change = 0.15).

The SEM approach to incremental validity demonstrated for the CFA that a unidimensional model did not exhibit satisfactory fit, χ^2^ (88, *N* = 5,305), CFI = 0.78, SRMR = 0.09, RMSEA = 0.10 (95% CI of 0.10 to 0.11). The two-dimensional model revealed improved fit, χ^2^ (87, *N* = 5,305), CFI = 0.94, SRMR = 0.04, RMSEA = 0.05 (95% CI of 0.05 to 0.06). Factor correlations demonstrated that aMTQ10 and SBS possessed a large positive association, *r* = 0.59. Incremental validity in the SEM method (i.e., testing partial regression coefficients in relation to SWLS) revealed that aMTQ10 significantly predicted SWLS in the presence of SBS. The unique predictive relationship was *ß* = 0.48 (*p* < 0.001), which was greater in magnitude than SBS (*ß* = 0.36, *p* < 0.001). Collectively, these findings indicate that the aMTQ10 explains unique variance in life satisfaction beyond belongingness and supports the discriminant validity of the aMTQ10 relative to related psychosocial constructs.

### Norm values

Tables 3–6 contain population norms including percent ranks and stanine values for the total sample as well as normative values stratified by gender, school year, and school type. Percent ranks indicate the percentage of pupils scoring at or below a given score, whereas stanines provide a standardized nine-point classification ranging from very low to very high scores.

**Table 3:** Percent rank and stanine values for the aMTQ10 in the total sample.

Total sample (*N* = 5,305)		
Mean scores	PR	Stanine
1.00	0.04	1.00
1.10	0.07	1.00
1.20	0.12	1.00
1.30	0.19	1.00
1.40	0.31	1.00
1.50	0.48	1.00
1.60	0.73	1.00
1.70	1.09	1.00
1.80	1.61	1.00
1.90	2.32	1.00
2.00	3.27	1.00
2.10	4.53	2.00
2.20	6.14	2.00
2.30	8.18	2.00
2.40	10.69	3.00
2.50	13.72	3.00
2.60	17.28	3.00
2.70	21.38	3.00
2.80	25.99	4.00
2.90	31.08	4.00
3.00	36.55	4.00
3.10	42.31	5.00
3.20	48.24	5.00
3.30	54.21	5.00
3.40	60.09	6.00
3.50	65.74	6.00
3.60	71.07	6.00
3.70	75.97	6.00
3.80	80.38	7.00
3.90	84.26	7.00
4.00	87.59	7.00
4.10	90.40	8.00
4.20	92.71	8.00
4.30	94.57	8.00
4.40	96.03	9.00
4.50	97.16	9.00
4.60	98.0	9.00
4.70	98.62	9.00
4.80	99.07	9.00
4.90	99.39	9.00
5.00	99.60	9.00

PR, percent rank.

**Table 4:** Percent ranks and stanine values for the aMTQ10 stratified by gender.

Total sample (*N* = 5,305)			Girls (*n* = 2,578)		Boys (*n* = 2,727)	
Mean scores	PR	Stanine	PR	Stanine	PR	Stanine
1.00	0.04	1.00	0.10	1.00	0.01	1.00
1.10	0.07	1.00	0.16	1.00	–	–
1.20	0.12	1.00	0.26	1.00	0.02	1.00
1.30	0.19	1.00	0.41	1.00	0.04	1.00
1.40	0.31	1.00	0.64	1.00	0.06	1.00
1.50	0.48	1.00	0.97	1.00	0.11	1.00
1.60	0.73	1.00	1.44	1.00	0.18	1.00
1.70	1.09	1.00	2.11	1.00	0.30	1.00
1.80	1.61	1.00	3.01	1.00	0.48	1.00
1.90	2.32	1.00	4.22	2.00	0.76	1.00
2.00	3.27	1.00	5.78	2.00	1.17	1.00
2.10	4.53	2.00	7.78	2.00	1.75	1.00
2.20	6.14	2.00	10.25	2.00	2.57	1.00
2.30	8.18	2.00	13.26	3.00	3.68	1.00
2.40	10.69	3.00	16.82	3.00	5.17	2.00
2.50	13.72	3.00	20.94	3.00	7.09	2.00
2.60	17.28	3.00	25.61	4.00	9.52	2.00
2.70	21.38	3.00	30.76	4.00	12.52	3.00
2.80	25.99	4.00	36.33	4.00	16.12	3.00
2.90	31.08	4.00	42.20	5.00	20.33	3.00
3.00	36.55	4.00	48.25	5.00	25.14	4.00
3.10	42.31	5.00	54.34	5.00	30.50	4.00
3.20	48.24	5.00	60.33	6.00	36.30	4.00
3.30	54.21	5.00	66.09	6.00	42.44	5.00
3.40	60.09	6.00	71.49	6.00	48.77	5.00
3.50	65.74	6.00	76.45	6.00	55.14	5.00
3.60	71.07	6.00	80.89	7.00	61.37	6.00
3.70	75.97	6.00	84.77	7.00	67.32	6.00
3.80	80.38	7.00	88.09	7.00	72.86	6.00
3.90	84.26	7.00	90.87	8.00	77.89	7.00
4.00	87.59	7.00	93.13	8.00	82.34	7.00
4.10	90.40	8.00	94.93	8.00	86.17	7.00
4.20	92.71	8.00	96.34	9.00	89.40	7.00
4.30	94.57	8.00	97.41	9.00	92.04	8.00
4.40	96.03	9.00	98.20	9.00	94.15	8.00
4.50	97.16	9.00	98.78	9.00	95.80	8.00
4.60	98.0	9.00	99.19	9.00	97.04	9.00
4.70	98.62	9.00	99.47	9.00	97.97	9.00
4.80	99.07	9.00	99.66	9.00	98.63	9.00
4.90	99.39	9.00	99.79	9.00	99.10	9.00
5.00	99.60	9.00	99.87	9.00	99.42	9.00

PR, percent rank. A dash (−) indicates that no participants within the subgroup obtained the corresponding mean score.

**Table 5:** Percent ranks and stanine values for the aMTQ10 stratified by year group.

Total sample (*N* = 5,305)			Year 9 (age 13–14) (*n* = 2,865)		Year 10 (age 14–15) (*n* = 2,440)	
Mean scores	PR	Stanine	PR	Stanine	PR	Stanine
1.00	0.04	1.00	0.01	1.00	0.10	1.00
1.10	0.07	1.00	–	–	0.16	1.00
1.20	0.12	1.00	0.03	1.00	0.26	1.00
1.30	0.19	1.00	0.04	1.00	0.41	1.00
1.40	0.31	1.00	0.08	1.00	0.64	1.00
1.50	0.48	1.00	0.13	1.00	0.98	1.00
1.60	0.73	1.00	0.22	1.00	1.46	1.00
1.70	1.09	1.00	0.36	1.00	2.12	1.00
1.80	1.61	1.00	0.57	1.00	3.03	1.00
1.90	2.32	1.00	0.88	1.00	4.23	2.00
2.00	3.27	1.00	1.34	1.00	5.80	2.00
2.10	4.53	2.00	1.99	1.00	7.80	2.00
2.20	6.14	2.00	2.88	1.00	10.27	2.00
2.30	8.18	2.00	4.09	2.00	13.27	3.00
2.40	10.69	3.00	5.69	2.00	16.82	3.00
2.50	13.72	3.00	7.73	2.00	20.94	3.00
2.60	17.28	3.00	10.29	2.00	25.59	4.00
2.70	21.38	3.00	13.42	3.00	30.74	4.00
2.80	25.99	4.00	17.14	3.00	36.29	4.00
2.90	31.08	4.00	21.47	3.00	42.15	5.00
3.00	36.55	4.00	26.37	4.00	48.18	5.00
3.10	42.31	5.00	31.78	4.00	54.26	5.00
3.20	48.24	5.00	37.62	4.00	60.24	6.00
3.30	54.21	5.00	43.75	5.00	65.99	6.00
3.40	60.09	6.00	50.04	5.00	71.39	6.00
3.50	65.74	6.00	56.32	5.00	76.35	6.00
3.60	71.07	6.00	62.45	6.00	80.79	7.00
3.70	75.97	6.00	68.29	6.00	84.68	7.00
3.80	80.38	7.00	73.69	6.00	88.01	7.00
3.90	84.26	7.00	78.59	7.00	90.79	8.00
4.00	87.59	7.00	82.91	7.00	93.06	8.00
4.10	90.40	8.00	86.62	7.00	94.88	8.00
4.20	92.71	8.00	89.74	8.00	96.29	9.00
4.30	94.57	8.00	92.30	8.00	97.37	9.00
4.40	96.03	9.00	94.33	8.00	98.17	9.00
4.50	97.16	9.00	95.92	8.00	98.76	9.00
4.60	98.0	9.00	97.13	9.00	99.17	9.00
4.70	98.62	9.00	98.02	9.00	99.46	9.00
4.80	99.07	9.00	98.67	9.00	99.65	9.00
4.90	99.39	9.00	99.12	9.00	99.78	9.00
5.00	99.60	9.00	99.43	9.00	99.87	9.00

PR, percent rank. A dash (−) indicates that no participants within the subgroup obtained the corresponding mean score.

**Table 6:** Percent ranks and stanine values for the aMTQ10 stratified by school type.

Total sample (*N* = 5,305)			Private school (*n* = 4,227)		State school (*n* = 1,078)	
Mean scores	PR	Stanine	PR	Stanine	PR	Stanine
1.00	0.04	1.00	0.03	1.00	0.14	1.00
1.10	0.07	1.00	–	–	0.21	1.00
1.20	0.12	1.00	0.08	1.00	0.33	1.00
1.30	0.19	1.00	0.14	1.00	0.50	1.00
1.40	0.31	1.00	0.22	1.00	0.75	1.00
1.50	0.48	1.00	0.36	1.00	–	–
1.60	0.73	1.00	0.56	1.00	1.59	1.00
1.70	1.09	1.00	0.86	1.00	2.24	1.00
1.80	1.61	1.00	1.29	1.00	3.12	1.00
1.90	2.32	1.00	1.89	1.00	4.25	2.00
2.00	3.27	1.00	2.71	1.00	5.70	2.00
2.10	4.53	2.00	3.82	1.00	7.52	2.00
2.20	6.14	2.00	5.27	2.00	9.74	2.00
2.30	8.18	2.00	7.13	2.00	12.41	3.00
2.40	10.69	3.00	9.46	2.00	15.56	3.00
2.50	13.72	3.00	12.30	3.00	19.19	3.00
2.60	17.28	3.00	15.69	3.00	23.30	4.00
2.70	21.38	3.00	19.64	3.00	27.86	4.00
2.80	25.99	4.00	24.15	4.00	32.81	4.00
2.90	31.08	4.00	29.16	4.00	38.08	4.00
3.00	36.55	4.00	34.61	4.00	43.58	5.00
3.10	42.31	5.00	40.40	5.00	49.21	5.00
3.20	48.24	5.00	46.41	5.00	54.86	5.00
3.30	54.21	5.00	52.51	5.00	60.41	6.00
3.40	60.09	6.00	58.54	5.00	65.76	6.00
3.50	65.74	6.00	64.38	6.00	70.80	6.00
3.60	71.07	6.00	69.90	6.00	75.47	6.00
3.70	75.97	6.00	75.00	6.00	79.71	7.00
3.80	80.38	7.00	79.60	7.00	83.48	7.00
3.90	84.26	7.00	83.65	7.00	86.76	7.00
4.00	87.59	7.00	87.14	7.00	89.56	8.00
4.10	90.40	8.00	90.08	8.00	91.91	8.00
4.20	92.71	8.00	92.49	8.00	93.83	8.00
4.30	94.57	8.00	94.43	8.00	95.38	8.00
4.40	96.03	9.00	95.95	8.00	96.59	9.00
4.50	97.16	9.00	97.11	9.00	97.54	9.00
4.60	98.0	9.00	97.98	9.00	98.25	9.00
4.70	98.62	9.00	98.62	9.00	98.78	9.00
4.80	99.07	9.00	99.08	9.00	99.16	9.00
4.90	99.39	9.00	99.39	9.00	99.44	9.00
5.00	99.60	9.00	99.61	9.00	–	–

PR, percent rank. A dash (−) indicates that no participants within the subgroup obtained the corresponding mean score.

## Discussion

This study developed and psychometrically evaluated the Adolescent Mental Toughness Questionnaire (aMTQ10). Analysis established that the aMTQ10 is a robust instrument for measuring global mental toughness (MT) in adolescents within secondary school and equivalent educational settings. Regarding the factor structure, confirmatory factor analysis established that the aMTQ10 replicated the one-factor correlated uniqueness model structure observed in the adult MTQ10 (Dagnall et al., 2019; Denovan et al., 2024). High internal reliability further attested to the measurement properties of the aMTQ10, and factor loadings indicated strong construct validity. This alignment was expected, as the aMTQ10 is derived from the MTQ suite of measures, which assess MT as defined by Clough’s 4/6C model.

While debates regarding the precise definition of MT persist, the widespread use and publication of studies employing MTQ measures demonstrate that they effectively assess core components of the construct (i.e., challenge, control, commitment, and confidence). These elements, alongside self-efficacy and emotion regulation, contribute to a positive mindset—a collection of enabling non-cognitive skills—that increases resistance to stress and enhances coping (Denovan et al., 2023a,b). These results indicate that the modifications made to the MTQ10 to enhance adolescent comprehension did not undermine the instrument’s psychometric integrity. Importantly, the relatively minor nature of the wording modifications suggests that the core MTQ10 framework is broadly applicable to adolescent populations, while still benefiting from developmental refinement to maximize accessibility and comprehension.

Invariance testing revealed partial gender invariance, indicating that while subtle differences existed in how boys and girls interpreted specific items, both groups interpreted the underlying construct of MT similarly. Full scalar invariance was established for school type (private vs. state) and year group (Year 9 vs. Year 10). Thus, the analysis confirmed the equivalence of the aMTQ10 across these subgroups, meaning that observed latent mean differences are attributable to true differences in MT rather than variations in scale interpretation.

In this context, girls scored significantly lower than boys. This outcome concurs with findings from both adolescent (Clough et al., 2008) and general samples (Ahsan et al., 2025). The present findings are also broadly consistent with prior adolescent MT research using the MTS-A, which similarly identified meaningful associations between MT and adaptive educational and psychological functioning (McGeown et al., 2018). An explanation is the observed decline in MT for girls during early-to-mid adolescence (ages 12–14), which typically increases as they progress through late adolescence (Jones, 2021). This trend is more pronounced in girls and is often associated with the complex developmental challenges of this period, including heightened social pressures, self-consciousness, and identity formation. Additionally, societal expectations and gender roles may influence girls to express emotions and seek support more openly, whereas boys may feel pressured to suppress vulnerability, leading to distinct gender-related perceptions of MT. Further research into these developmental trajectories is required to explore these possibilities more fully.

Additionally, Year 10 pupils (aged 14–15) scored lower on MT than Year 9 pupils (aged 13–14). This difference may be explained by the transition from Key Stage 3 to Key Stage 4, a demanding period as students shift focus from a broader curriculum to specialized GCSE courses and formal qualifications. This change in emphasis can be stressful and disorienting (Denovan and Dagnall, 2023). This supposition is consistent with research demonstrating that major educational transitions (e.g., primary to secondary school, Sirsch, 2003; or college to university, Denovan et al., 2020) and the heightened awareness of high-stakes assessments are significant sources of anxiety in educational contexts (Putwain, 2008).

Lastly, state school pupils scored lower on the aMTQ10 than their private school peers. This outcome aligns with the Independent Schools Council report, Understanding “Soft Skills” Development at Independent Schools: An Analysis of Mental Toughness at UK Independent Schools (AQR, 2017). This difference is attributable to socio-economic and environmental factors, suggesting that students in different educational sectors face varying pressures and have differing access to psychological resources and support systems. This aligns with the view of MT as a malleable psychological resource that can be developed through experience and intervention (i.e., a plastic trait). This is particularly true for the dimensions of control and commitment, which are less linked to heritability (Dagnall et al., 2019; Denovan et al., 2023a). Subsequent research should investigate the specific drivers of these differences between state and private schools, as scholarly work in this area remains underdeveloped.

From a measurement perspective, the establishment of partial invariance and the alignment of these findings with preceding studies indicate that the aMTQ10 possesses strong construct validity and is an appropriate age-adapted scale for adolescents. Incremental validity findings support this; the aMTQ10 uniquely contributed to the prediction of life satisfaction even after accounting for sense of belonging. Moreover, structural equation modeling (SEM) highlighted that the aMTQ10’s unique predictive relationship with life satisfaction was greater than that of belonging, a result consistent with previous research using the MTQ10 (Dagnall et al., 2019). This suggests that the aMTQ10 captures a distinct facet of well-being with significant explanatory power. In practical terms, this indicates that MT contributes uniquely to adolescent well-being beyond the influence of social connectedness and belonging.

A significant practical contribution of this study is the production of a comprehensive set of adolescent aMTQ10 population norms based on gender, school year, and school type. These norms provide standardized benchmarks for researchers and practitioners to interpret individual scores. Rather than relying on raw scores, investigators can now determine whether an individual’s score is low, average, or high relative to their peers. These benchmarked scores enable informed educational and clinical decision-making, such as identifying individuals who would benefit most from targeted interventions to nurture MT. Potential interventions may include resilience-based educational programs, psychological skills training, stress-management interventions, and school-based wellbeing initiatives designed to enhance confidence, emotional regulation, and adaptive coping. Consequently, these norms afford a more nuanced and contextually relevant understanding of MT during adolescence.

### Limitations and suggestions for future research

Despite these findings, it is important to acknowledge study limitations. First, the sample comprised exclusively students from UK secondary schools. This restricts the generalizability of the results, particularly the norms, to adolescents from other educational and cultural contexts. To extend this study, investigators should assess independent samples from distinct cultures and perform further age-appropriate adaptations. These efforts could inform the establishment of norms for other educational groups, such as primary school pupils and students in further or higher education. While young adults and university students can complete the standard MTQ10, the generation of additional subgroup norms would facilitate a deeper understanding of their levels of MT relative to their peers.

While the present paper demonstrates the psychometric robustness of the aMTQ10, future scholarly work should further assess the scale’s measurement properties. Particularly, research should examine convergent and discriminant validity with a range of psychological constructs pertinent to adolescent development, such as identity formation, self-esteem, emotional regulation, and academic motivation. Ensuring that the aMTQ10 correlates appropriately with conceptually similar and dissimilar constructs will further attest to the validity of the instrument.

Additionally, because this study used a cross-sectional design to compare independent samples of Year 9 and Year 10 pupils, researchers should conduct longitudinal studies to enable the evaluation of within-student changes. Such comparisons would highlight how educational transitions affect MT and establish the predictive utility of the construct for adolescent outcomes, including academic trajectories and mental health. Relatedly, multiple time-point testing would provide evidence of aMTQ10 stability via test–retest reliability.

Within these longitudinal designs, investigators also need to consider the contextual factors contributing to observed group differences. This includes examining the influence of the school curriculum, peer dynamics, and the family environment to provide a more holistic understanding of how MT is nurtured or hindered during adolescence.

## Data Availability

The raw data supporting the conclusions of this article will be made available by the authors, without undue reservation.
