# Supplementary material for: Adolescent Mental Toughness Questionnaire (aMTQ10): development, validation and norms
Source: Front Psychol. 2026 Jun 23;17:1661207. doi: 10.3389/fpsyg.2026.1661207 (PMC13337688; doi:10.3389/fpsyg.2026.1661207)
Supplement: Supplementary file 1 [file Table_1.DOCX]

Supplementary Table S1. Original MTQ10 items, adolescent-adapted aMTQ10 items, and developmental wording modifications

| Original MTQ10 Item | aMTQ10 Item | Modification |
| --- | --- | --- |
| Even when under considerable pressure I usually remain calm | Even when under lots of pressure I usually remain calm | “considerable pressure” simplified to “lots of pressure” |
| I tend to worry about things well before they actually happen | I tend to worry about things well before they actually happen | Unchanged |
| I usually find it hard to summon enthusiasm for the tasks I have to do | It is usually hard for me to find enthusiasm for the tasks I have to do | Simplified phrasing (“summon enthusiasm” removed) |
| I generally cope well with any problems that occur | I generally cope well with any problems that occur | Unchanged |
| I generally feel that I am a worthwhile person | I generally feel that I am a worthwhile person | Unchanged |
| “I just don’t know where to begin” is a feeling I usually have when presented with several things to do at once | “I just don’t know where to begin” is a feeling I usually have when given several things to do at once | “presented with” simplified to “given” |
| When I make mistakes, I usually let it worry me for days after | When I make mistakes I usually let it worry me for days after | Minor punctuation change to reduce syntactic complexity |
| I generally feel in control | I generally feel in control | Unchanged |
| I am generally able to react quickly when something unexpected happens | I am generally able to react quickly when something unexpected happens | Unchanged |
| I generally look on the bright side of life | I generally look on the bright side of life | Unchanged |
